# Validity of the diagnosis of a single depressive episode in a case register

**DOI:** 10.1186/1745-0179-5-4

**Published:** 2009-02-12

**Authors:** Camilla Bock, Jens Drachmann Bukh, Maj Vinberg, Ulrik Gether, Lars Vedel Kessing

**Affiliations:** 1 Department of Psychiatry, Rigshospitalet, University Hospital of Copenhagen, Copenhagen, Denmark; 2 Center for Pharmacogenomics, Department of Neuroscience and Pharmacology, Faculty of Health Sciences, University of Copenhagen, Copenhagen, Denmark

## Abstract

**Objective:**

To validate the ICD-10 diagnosis of a single depressive episode as used in daily clinical psychiatric practice and as recorded in the Danish Psychiatric Central Research Register.

**Methods:**

Patients discharged with a diagnosis of a single depressive episode were consecutively sampled from the register and diagnosed according to an interview using the Schedules for Clinical Assessment in Neuropsychiatry (SCAN).

**Results:**

A total of 75.4% of 399 patients with a register diagnosis of a single depressive episode also got this diagnosis according to the SCAN interview (82.8% for severe type of a single depression, 76.0% for moderate type of a single depression and 65.2% for mild type of a single depression).

**Conclusion:**

The ICD-10 diagnosis of a single depressive episode can be used in daily clinical practice with sufficient precision. The validity of the diagnosis is highest for severe and moderate type of depression and decreases for mild depression.

## Significant outcomes

• Clinicians seem to use the ICD-10 diagnosis of a single depressive episode in daily clinical practice with sufficient precision.

• The validity of the ICD-10 diagnosis of a single depressive episode, as recorded in the Danish Psychiatric Central Research Register, seems reasonably good.

• The validity of the diagnosis is highest for severe and moderate type of depression and decreases for mild depression.

## Limitations

• The validity of diagnostic subtypes of a single depressive episode was low.

• Only patients with a Danish origin and who took an antidepressant drug for at least one week were included in the study.

• Researchers were not blinded for the clinical diagnosis.

## Introduction

Psychiatric case registers has been increasingly used both in research and in mental health planning in general [1-5]. Within affective disorder several studies have used data from case registers [6-8] including the Danish Psychiatric Central Research Register, DPCRR [9-14].

Nationwide population based case registers such as the DPCRR offer evident advantages for research purposes as data are easy accessible at a very low cost. Further, since data are very accurately registered for nearly 100% of the Danish population, the data are not subject to selection bias. Recall bias is omitted because data are registered as part of daily clinical routine and independently of researchers and specific studies.

As case registers include data on large populations over many years or even decades, such data offer extraordinary possibilities for longitudinal research on rare conditions such as completed suicide [15,16] or on repeated outcomes, such as readmission rates over many years [17,18] or changes in diagnosis over decades [19,20]. Such studies would be difficult or even impossible to undertake validly in other ways, since loss to follow-up often would be pronounced due to simple drop-out of the study or to incomplete registration of death or migration (e.g., 19% in the 16 years Zurich follow-up study [21] and 26% in the six years follow-up study by the National Institute of Mental Health (NIMH) – Clinical Research Branch Collaboratory Program on the Psychobiology of Depression [22]). In contrast, loss to follow-up is negligible in the Danish case register because this covers all Danish treatment institutions and since registration of death and migration is virtually complete in Denmark [23]. Finally, it would be practically and/or economically hazardous to conduct large scale studies on large samples of patients who should be followed for many years or even decades.

Nevertheless, case register based research is hampered by other disadvantages such as the use of clinical diagnoses that are not research based, selection of patients with the more severe kind of disorders who gain contact to the hospital health care system as in- or outpatients and the lack of data on treatment or other variables of possible interest or confounding effects.

To the best of our knowledge, the validity of the ICD-10 diagnosis of a single depressive episode as given in daily clinical practice has newer been investigated. Similarly, these diagnoses, as recorded in case registers, have never been validated. Hence, considering the widely use of the DPCRR in psychiatric research there is a need for validating the register diagnosis as recently recommended [24,25].

The aim of the present study was to validate the ICD-10 diagnosis of a single depressive episode as used in daily clinical practice in psychiatric in- or outpatient hospital settings in Denmark and as recorded in the DPCRR as compared to the diagnoses given at a research interview using the Schedules for Clinical Assessment in Neuropsychiatry (SCAN, [26]). We defined validity as the degree to which data represents that which it purports to and investigated the accuracy of the source of information itself in accordance with the definitions by Byrne et al [24]. As it is the clinical impression that patients who gain contact to psychiatric hospital settings due to depression frequently suffer from comorbid personality disorders we chose to characterize the sample further assessing the prevalence of comorbid personality disorder using The Structured Clinical Interview for DSM – IV Axis II Disorders (SCID II) [27]. We evaluated the register diagnosis in an aggregated manner (a single depressive episode) and according to the different diagnostic subtypes – (single depressive episode, unspecified, mild, moderate or severe). The study was part of a larger ongoing study investigating genetic and non-genetic predictors of treatment response to antidepressants.

## Methods

### The register

The DPCRR is nation-wide with registration of all psychiatric hospitalisations in Denmark for the 5.4 million inhabitants [28]. From January 1, 1995 the register included information on patients in psychiatric ambulatories and community psychiatry centres, also. All inhabitants in Denmark have a unique person identification number (Civil Person Registration number, CPR-number) that can be logically checked for errors, and, thus it can be established with great certainty if a patient previously has had contact to psychiatric service, irrespective of changes in name etc.

In the DPCRR, routine data validation is restricted to include identity of the patient, dates of admission and discharge, and correspondence between the written diagnosis from the case sheet and the coded diagnosis from the reported data [28].

The diagnoses registrated in the DPCRR are made by different clinicians all over Denmark and to improve the diagnostic reliability among clinicians, Danish specialists in psychiatry have completed courses in ICD-10.

No private psychiatric inpatient hospitals or department are in operation in Denmark, all are organised within public services and reporting to DPCRR. Private practising psychiatrists treat approximately 15.000 patients on a yearly basis, not reported to the DPCRR. The International Classification of Diseases, 10th Revision [29] has been used in Denmark from January 1, 1994.

### The sample

The study sample was defined as all outpatients (patients in psychiatric ambulatories and community psychiatry centres) and inpatients (patients admitted during daytime or overnight to a psychiatric hospital) with a diagnosis of a single depressive episode (ICD-10, code DF32-32.9) at the end of first contact ever. Patients were sampled consecutively every second month from the DPCRR in a period from 2005 June 1 to 2007 June 1 aiming at including patients in the study shortly after they were recorded in the register with a diagnosis given at discharge from hospital or out-patient contact. To improve participation in the interview only patients currently living in the eastern part of Denmark (Zealand (Sjælland)) were sampled from the register. Patients were invited to participate in the study by an invitation letter, and those who did not respond received an invitation again some months later. Patients who remained unresponsive were attempted contacted by telephone.

As the study was part of a larger study on the effect of genetic and nongenetic predictors of medical treatment outcome, patients were excluded from interview if they were not of Danish ethnicity (proband and their parents born in Denmark and grandparents born in Europe), or if they only received treatment with an antidepressant for the depressive episode for less than one week.

### Diagnostic interview

SCAN diagnoses were obtained for the current episode leading to psychiatric contact and for lifetime before the current episode and was based on the interview with the patients and data from their psychiatric hospital case notes. The period from psychiatric discharge to the time of the interview was disregarded.

The Structured Clinical Interview for DSM – IV Axis II Personality Disorders (SCID-II) was made to assess the prevalence of personality disorders [27].

Diagnoses were obtained by two medical doctors (CB and JDB) who had followed a WHO-certified course in the use of the SCAN interview. The two doctors conducted 10 co-ratings on patients in a pilot phase prior to start of the study and every second month during the 2-year inclusion period of the study, resulting in 26 co-ratings. During these co-ratings, the two medical doctors alternated to do the SCAN and SCID interviews and rated the patients independently following the interview. The kappa for interrater agreement on the diagnosis of a single depressive episode (yes/no) was 1.00 and for a diagnosis of personality disorder (yes/no) was 0.76.

Further, patients were rated on the Hamilton Depression Scale, 17 items [30] and fulfilled 21-items Beck Depression Inventory [31] besides a large number of other scales and questionnaires.

### Ethics

The Danish ministry of health, The Danish Ethic Committee ((KF) 01.209/04) and the Data Inspection approved the study. The investigation was conducted in accordance with the latest version of the Declaration of Helsinki. All participants gave written informed consent.

### Statistical analysis

Categorical data were analysed with chi-square test (2-sided) and continuous data were analysed with the Mann-Whitney test for two independent groups. P < 0.05 was used to indicate statistical significance. SPSS software package for windows, version 13.0 was used [32].

## Results

A total number of 1486 patients with a diagnosis of a single depressive episode at first in- or outpatient contact were sampled from the register. Among the 1486 patients, 480 were excluded (data protection in relation to research (N = 78), not Danish ethnicity (N = 291), had not taken antidepressants for more than a week (N = 78), did not live at Sealand (N = 10), had a major physical handicap that made participation impossible, suffered from dementia or oligophrenia (N = 14) or had died following discharge from hospital treatment (N = 9)). The remaining 1006 patients were invited to participate in the interview but 607 patients refused to participate leaving 399 patients for the face-to-face interview.

As can be seen from Table 1, the 399 participants in the study did not differ from the 1087 non-participants in age at first contact, gender, subtype of depression (mild, moderate, severe), or the duration of contact. However, significantly more in-patients than out-patients participated in the study. The 399 patients who participated in the interview had a mean score of 9.3 (SD: 6.2) on the Hamilton Depression Scale, 17 items [30] and a score of 15.3 (SD: 9.9) on the 21-item Beck Depression Inventory [31] at the time of the interview.

**Table 1:** Differences between participants and non-participants in the study based on register data.

	Participants in the interview	Non-participants in the interview	P
Number	399	1087	

Median age at first contact (quartiles)	38.2 (29.5–51.9)	39.6 (29.3–53.1)	0.7*

Gender			
Men (%)	140 (35.1)	417 (38.4)	0.2**
Women (%)	259 (64.9)	670 (61.6)	

Type of depression (ICD-10, %)			
Unspecified	43 (10.8)	128 (11.8)	
Mild	65 (16.3)	195 (17.9)	0.8**
Moderate	204 (51.1)	525 (48.3)	
Severe	87 (21.8)	239 (22.0)	

Type of contact (%)			
Admission	232 (58.1)	558 (51.3)	0.02**
Out-patient	167 (41.9)	529 (48.7)	

Duration of contact (quartiles)			
Admission in days	21.5 (6.0–45.0)	16.0 (4.0–41.0)	0.09*
Out-patient in days	70.0 (19.0–188.0)	88.0 (29.5–210.5)	0.1*

* Mann-Whitney test (25 – 75 percentiles).

** χ2 test.

Table 2 shows the main lifetime diagnoses from the discharge date and backward according to the SCAN interview in relation to the register main diagnoses, divided into single depressive episode total, mild, moderate and severe. The time interval from discharge to interview was on average 169.6 days (SD: 87.0). Among the 399 patients who got a diagnosis of a single depressive episode in the register, 301 patients also got a diagnosis of a single depressive episode according to the SCAN interview, corresponding to 75.4% accordance (second column). Sub-analyses according to gender showed that, overall, the diagnosis of a single depressive episode in the register was confirmed for 76.8% of women and for 72.9% of men according to the SCAN interview (p = 0.4).

**Table 2:** Case register main diagnoses in relation to main diagnoses according to Schedules for Clinical Assessment in Neuropsychiatry (SCAN).

Main diagnoses SCAN (research-based)	Main diagnoses case register (clinically based)				
	Single depressive episode totally	Single depressive episode unspecified	Single depressive episode mild	Single depressive episode moderate	Single depressive episode severe

Total	399	43	65	204	87

Schizophrenia, etc. F20-29 (%)	4 (1.0)	0 (0.0)	0 (0.0)	2 (1.0)	2 (2.3)

Bipolar disorder, F30-31 (%)	13 (3.3)	2 (4.7)	3 (4.6)	7 (3.4)	1 (1.1)

Single depressive episode, F32 (%)	301 (75.4)	31 (72.1)	43 (65.2)	155 (76.0)	72 (82.8)
mild	73 (18.3)	12 (27.9)	9 (13.6)	43 (21.1)	9 (10.3)
moderate	161 (40.4)	18 (41.9)	25 (37.9)	80 (39.2)	38 (43.7)
severe	67 (16.8)	1 (2.3)	9 (13.6)	32 (15.7)	25 (28.7)

Recurrent depressive disorder, F33 (%)	44 (11.0)	4 (9.3)	9 (13.6)	22 (10.8)	9 (10.3)

Dystymia, F34 (%)	2 (0.5)	0 (0.0)	1 (1.5)	1 (0.5)	0 (0.0)

Other (no diagnosis or psychoactive substance use (F.00-09), anxiety, stress-related and somatoform disorders (F40-49)) (%)	35 (8.8)	6 (14.0)	9 (13.6)	17 (8.3)	3 (3.4)

The most prevalent SCAN main diagnoses differing from single depressive episode were recurrent depressive disorder (11.0%), followed by "other" main diagnoses (8.8%), comprising mainly anxiety disorders (4.0%), eating and somatoform disorders (2.0%) as well as substance abuse including alcohol (2%), and finally few patients received no psychiatric diagnosis at all. Some of the patients related to the group labelled "other" diagnoses obtained more than one psychiatric diagnosis, and 37.1% of this group also received a diagnosis of any kind of personality disorder. Finally, 1.0% got a SCAN diagnosis of schizophrenia or non organic unspecified psychosis and 3.3% a diagnosis of bipolar disorder.

As can be further seen from Table 2, among the patients who were diagnosed with a single depressive episode, unspecified type in the register, the proportion of patients with a SCAN diagnosis of a single depressive episode was 72.1%. For patients with a single depressive episode, mild, moderate or severe in the register, the corresponding proportion of patients who got a diagnosis of a single depressive episode at the SCAN interview increased from 65.2% to 76.0% and to 82.8%.

A SCAN main diagnosis of schizophrenia (N = 3) or non organic psychosis (N = 1), respectively, was only given to patients with register diagnoses of the moderate or severe subtype of depression, whereas a diagnosis of bipolar disorder and also "other" diagnoses were more prevalent among patients with a register diagnosis of the unspecified or mild type of depression.

Table 3 shows the diagnoses according to The Structured Clinical Interview for DSM – IV Axis II Personality Disorders (SCID-II). In total 33.1% of patients participating in the interview obtained a diagnosis of personality disorder of any kind. Patients receiving a diagnosis of unspecified or mild single depressive episode for the first time according to the Psychiatric Central Research Register had a highly significant increased prevalence of comorbid cluster B personality disorders (including histrionic, narcissistic, antisocial and borderline personality disorders) according to the SCID II interview. Likewise, the prevalence of comorbid personality disorders of any kind was significantly higher among patients with a diagnosis of unspecified or mild depression compared to moderate and severe depression.

**Table 3:** Case register main diagnoses in relation to comorbid personality disorders according to The Structured Clinical Interview for DSM – IV Axis II Personality Disorders (SCID-II).

			Main diagnoses in case register			
	Single depressive episode, totally	Single depressive episode unspecified	Single depressive episode mild	Single depressive episode moderate	Single depressive episode severe	P*

Any Personality Disorder (%)	132 (33.1)	23 (53.5)	28 (43.1)	61 (29.9)	20 (23.0)	0.001

Cluster A (%)	15 (3.8)	3 (7.0)	0 (0.0)	11 (5.4)	1 (1.1)	0.08

Cluster B (%)	52 (13.0)	12 (27.9)	17 (26.2)	18 (8.8)	5 (5.7)	< 0.001

Cluster C (%)	70 (17.5)	11 (25.6)	10 (15.4)	35 (17.2)	14 (16.1)	0.5

Depressive Personality Disorder (%)	32 (8.0)	7 (16.3)	4 (6.2)	13 (6.4)	8 (9.2)	0.2

* χ2 test.

Cluster A includes paranoid, schizoid and schizotypal personality disorder.

Cluster B includes histrionic, narcissistic, antisocial and borderline personality disorder.

Cluster C includes avoidant, dependent and obsessive-compulsive personality disorder.

## Discussion

We found a rather high validity of the diagnosis of a single depressive episode in the DPCRR as the diagnosis was confirmed in 75.4% of the patients according to a SCAN interview. The validity of the register diagnosis was highest for patients with a severe single depressive episode, among whom 82.8% got a SCAN diagnosis of a single depressive episode. The validity decreased successively with regard to milder depressions, since 76.0% of patients with a moderate single depression and 65.2% of patients with a mild single depression obtained a SCAN diagnosis of single depressive episode. We have previously shown that the ICD-10 categorisation of a single depressive episode into mild, moderate and severe subtypes predicted the rate of relapse and the rate of suicide during six years of follow-up – with increasing rates from mild to moderate to severe depressive episodes [33]. Together with the finding of the rather good diagnostic validity found in the present study, these results indicate that the ICD-10 diagnosis of a single depressive episode can be used in daily clinical practice with sufficient precision. It should on the other hand be noted that the validity of diagnostic subtypes of a single depressive episode was low (the agreement between the case register diagnosis and the research diagnosis for severe episode = 28.7%, for moderate episode = 39.2% and for mild episode = 13.6%, see Table 2).

We did not take auxiliary diagnoses (co-morbid diagnoses) from the DPCRR into account since it is well known that such diagnoses are underreported by clinicians to the register [34]. However, we did examine the prevalence of co-morbid personality disorder at the face-to-face interview by use of the Structured Clinical Interview for DSM-IV Axis II and found that 33.1% of patients treated in psychiatric hospital settings for a single depressive episode had a co-morbid personality disorder. Further, we found a significantly higher prevalence of cluster B disorders in patients with a mild or unspecified single episode of depression compared to patients with a moderate or severe depression. The association between severity of depression and the prevalence of personality disorders has rarely been studied. In contrast to our results, two other studies have found a positive association between increasing severity of depression at the index episode and the prevalence of a personality disorder [35,36], specifically of the borderline, dependent and depressive personality disorder, but not of the avoidant type [35]. The discrepancy between our finding and findings from prior studies may be due to different recruitment procedures, populations in the studies (clinical sample recruited via advertisement [35], population-based non-clinical sample [36]) and the fact that we included patients with a single depressive episode only, in contrast to prior studies including mixed samples of single and recurrent depressions.

In relation to the DPCRR, a register diagnosis of a single episode of depression, mild or unspecified type, was only classified as a single depressive episode by SCAN interviews in 72.1% and 65.2% of the cases, respectively, (Table 2), and this relatively poor validity of the subdiagnosis of a single depressive episode, unspecified or mild type, may be due to the higher prevalence of personality disorders among these patients.

### Limitations

It is a limitation that researchers in the study were not blinded for the clinical/register diagnosis. Blinding of diagnosis is difficult to obtain throughout a 3–4 hour interview, as patients will tend to tell their diagnosis. Further, it can be argued that the validity of the research diagnosis becomes higher when researchers have access to all available information including case notes.

It should be stressed that the present study was part of a larger study on genetic and non-genetic predictors of response to antidepressants and therefore only patients with a Danish origin and patients who took an antidepressant drug for at least one week were interviewed. Besides that more in-patients than out-patients participated in the face-to-face interview, participants did not differ from non-participants on a wide range of variables, such as age at first contact, gender, register subtype of depression (mild, moderate, severe), or the duration of contact. The agreement between the register diagnosis of a single depressive episode and the SCAN diagnosis of a single depressive episode did not differ for men and women (72.9% versus 76.8%, p = 0.4). Taking together these data suggest that our results are not biased due to non-participation in the study, and that the findings can be generalized to all Caucasian patients with a diagnosis of a single depressive disorder in psychiatric in-and outpatients health care settings in Denmark. Whether our findings also can be generalised to psychiatric in-and outpatients health care settings in other countries is less clear as medical education, diagnostic training, etc. may vary between countries. It should be stressed that our findings cannot be generalized to patients with milder forms of depression who are treated in primary care or by private specialists in psychiatry.

### Advantages

Our study has several advantages. We received information from the DPCRR on all patients recorded in the register during a period of two years and invited all who met inclusion criteria (ethnic Danes, aged between 18 and 70 years and living in Sealand). We conducted SCAN interviews on a large sample of 399 patients. Recall bias due to the prevalence of more severe depressive symptoms at the time of interview, was reduced as patients had a mean score of 9.3 (SD: 6.2) on The Hamilton Depression Scale, only, following discharge from in- or outpatient treatment. The patients were sampled consecutively every second month from the register resulting in a mean latency period of just less than half a year.

To the best of our knowledge the ICD-10 diagnosis of a single depressive episode as given in daily clinical practice has never been investigated before. Taimenen et al compared clinical DSM-IV first-admission discharge diagnosis in a sample of 116 patients with psychosis, bipolar disorder or severe major depression with best estimate research DSM-IV diagnoses [37]. Although the overall diagnostic agreement was moderate, the diagnostic agreement was higher for severe major depression with a kappa value 0.71 (95% CI: 0.59–0.83). In general, the diagnostic agreement between clinical and research diagnoses has been found higher for schizophrenia and bipolar disorder [38].

We have previously validated the ICD-8 diagnoses of manic-depressive psychosis at first admission and found that among a random subsample from the Danish Psychiatric Central Register of 100 patients with a clinical ICD-8 diagnosis of manic-depressive psychosis at first admission, 95 patients received a life-time ICD-10 diagnosis of affective disorder according to research diagnostic criteria [39]. This figure is high also compared to the finding of an agreement between register diagnosis and the SCAN diagnosis of 75.4% in the present study, probably reflecting the more severe and narrow defined ICD-8 diagnosis of manic-depressive psychosis compared to the ICD-10 concept of a single depressive episode. West et al examined the validity of ICD-9 depression diagnoses recorded in a Canadian case register by use of case notes and prescription of antidepressant medication. Thus patients were not diagnosed according to a research based diagnostic interview. In accordance with our results, an agreement of 77% and 75.5% between the register and the case notes/prescription of antidepressants regarding depression without, respectively with anxiety [40]. Apart from the two studies mentioned above, no other studies have investigated the validity of affective disorder diagnoses recorded in psychiatric case registers.

In conclusion, the ICD-10 diagnosis of a single depressive episode can be used in daily clinical practice with sufficient precision. The validity of the diagnosis is highest for severe and moderate type of depression and decreases for mild depression.

## Competing interests

The authors declare that they have no competing interests.

## Authors' contributions

CB and LVK managed the literature searches. CB wrote the first draft of the manuscript. JDB, CB, and LVK undertook the statistical analyses. All authors participated in the process of designing the study. All authors contributed to and have approved the final manuscript.
